# In Reply to “Fertility Preservation in Young Adult Patients With Rectal Cancer: A Few Things to Consider”

**DOI:** 10.1093/oncolo/oyac149

**Published:** 2022-08-16

**Authors:** Julia Stal, Afsaneh Barzi, Kimberly A Miller

**Affiliations:** Department of Population and Public Health Sciences, Keck School of Medicine of USC, Los Angeles, CA, USA; Department of Medical Oncology and Therapeutics Research, City of Hope National Medical Center, Duarte, CA, USA; Department of Population and Public Health Sciences, Keck School of Medicine of USC, Los Angeles, CA, USA; Department of Dermatology, Keck School of Medicine of the University of Southern California, Los Angeles, CA, USA

## Abstract

This letter to the editor continues the discussion on fertility counseling for young adult patients with rectal cancer.

We thank Dr. Zhou for his comments on our study.^1,2^ As pointed out by Dr. Zhou, inclusion of patients younger than the age of 50 years is important given the significance of limitations in reproductive age. Although males have a greater reproductive timeframe than females (females, before mid-40s; males, <60s, as defined by the American Society for Reproductive Medicine [ASRM]), 50 years is considered an acceptable standard and used in other studies.^3-7^ Patient age serves as a primary determining factor for the need for fertility discussions. While clinical factors, such as disease stage, may inform the speed with which treatment must begin, all patients of reproductive age should receive a comprehensive fertility discussion tailored to their future reproductive status.

From a clinical perspective, rectal cancer treatment is moving toward a more aggressive course of multimodal therapy. In the past, radiation or chemo-radiation was the first line of treatment, followed by surgery and adjuvant therapy. Based on reported trials over the past several years, many providers are now beginning rectal cancer treatment with total neoadjuvant therapy, which delivers both chemotherapy and neoadjuvant chemoradiotherapy.^8^ As improvements in pathologic complete response have been reported, we can expect the 5-year relative survival rate for rectal cancer to increase over time (67% based on patients diagnosed from 2011 to 2017).^9,10^ However, more aggressive treatment regimens, such as total neoadjuvant therapy, increases the risk of iatrogenic infertility. As such, fertility discussions are likely to become more pertinent than ever in this patient population.

While we agree that patients with localized stage cancer who only need surgery for treatment may not have a threat to their fertility, the likelihood of diagnosis of localized cancer in patients younger than 50 years is notably lower than in patients aged 50 years or older, and the likelihood of locoregional stage where multimodality treatment is needed is much higher in this population.^11^

Fertility discussions can be equally as important as cancer treatment in preserving patient well-being and ensure that patients have the ability to make informed decisions about their reproductive futures. Therefore, the value of timely, comprehensive fertility discussions should not be underestimated. We appreciate Dr. Zhou’s further comments on the need for oncofertility tailored to patient sex and believe that addressing gaps in patient fertility counseling will support high quality of life for young survivors of rectal cancer.
